# Modelling excess mortality from non-communicable diseases during wartime: application to the Gaza Strip, occupied Palestinian territories

**DOI:** 10.1186/s12963-025-00426-5

**Published:** 2025-11-26

**Authors:** Hanan Abukmail, Zhixi Chen, Zeina Jamaluddine, Sarah Aly, Takeru Igusa, Paul B. Spiegel, Francesco Checchi

**Affiliations:** 1 Faculty of Epidemiology and Population Health, London School of Hygiene and Tropical Medicine, Keppel St, London, WC1E 7HT UK; 2 International Health System Research Group, Department of Engineering, University of Cambridge, Cambridge, UK; 3 Department of Emergency Medicine, Loma Linda University Medical Center, Loma Linda, CA 92354 USA; 4 Department of Civil and Systems Engineering, Johns Hopkins Whiting School of Engineering, Baltimore, MD 21218 USA; 5 Department of International Health, Center for Humanitarian Health, Johns Hopkins Bloomberg School of Public Health, Baltimore, MD 21205 USA

## Abstract

**Background:**

Patients with non-communicable diseases (NCDs) face multiple risks of excess mortality during wars. The Gaza Strip’s health services have been severely disrupted by Israel’s campaign since October 2023. We developed a modelling approach to project NCD excess mortality under three defined scenarios.

**Methods:**

We projected excess mortality from cancer (lung, colorectal, and breast), cardiovascular disease, diabetes mellitus type 1 patients, and chronic kidney disease requiring haemodialysis from February 2024 to August 2024. We defined three scenarios of treatment coverage: (i) ceasefire, (ii) status quo, and (iii) escalation. We used pre-war incidence and prevalence data to probabilistically simulate deaths among patient cohorts exposed to varying time-dependent mortality depending on their treatment status. We subtracted the expected non-crisis mortality based on pre-war data to compute excess deaths.

**Results:**

We projected 1,680, 2,480 and 2,680 excess deaths under the ceasefire, status quo and escalation scenarios, respectively, from February till August 2024, plus 1489 in the war’s earlier phase. Most deaths were projected among individuals aged >50 years old and from ischaemic heart disease.

**Conclusion:**

To our knowledge this is the first attempt to project NCD mortality in a live war setting, demonstrating potential impacts on NCD burden, particularly due to cardiovascular causes. The model focusses only on a subset of NCDs and neglects the impact of the crisis on disease progression, thereby plausibly underestimating actual mortality. It could inform better humanitarian resource allocation and service planning but requires refinement and improved parameterisation.

**Supplementary Information:**

The online version contains supplementary material available at 10.1186/s12963-025-00426-5.

## Introduction

Mortality is a key downstream indicator of the health impact of a crisis [1]. Excess mortality in crises triggered by war and natural disasters may be defined as deaths that are above and beyond those that would occur without the crisis [2]. This excess is primarily attributed to the disruption of health services and greater environmental exposure to risk factors [3].

In humanitarian crisis settings, such as war zones, healthcare disruption substantially affects people living with non-communicable diseases (NCDs) [4, 5]. NCD patients face risks of acute complications and accelerated disease progression [5]. These risks are attributed to reduced access to essential medicines, disrupted prevention programmes, worsening mental health, lack of healthy food options, and limited physical activity [6, 7], all of which are in turn exacerbated by forced displacement. Other studies have reported that acute NCD complications, for instance myocardial infarction, were higher in war settings and after natural disasters than under non-crisis conditions [8, 9].

Following Hamas attacks on Israel, the Gaza Strip has been under a large-scale military campaign since October 2023, which has caused a widespread health crisis. According to a United Nations report, Gaza’s healthcare facilities have been systematically targeted by the Israeli military [10]. As of December 2024, the World Health Organization had documented 617 attacks on health facilities since October 7, 2023, and no pre-existing health facility was fully operational. There have been numerous deaths, injuries and detentions among healthcare workers themselves [11]. In addition, Israel has imposed a blockade of Gaza, disrupting the flow of aid and the medical supply chain [12, 13]. This has led to a severe disruption of health services for NCD patients. For example, due to the destruction and siege of hospitals, many chronic kidney disease (CKD) patients have been unable to continue their haemodialysis treatment, while insulin shortages have put insulin-dependent diabetes mellitus (DM) patients at acute risk [14]. Reports also indicate the destruction of facilities offering renal dialysis and cancer care, and the interruption of pre-war medical and urgent referrals outside the Gaza Strip [15–18].

In many low- and middle-income countries, the reporting of NCD cases and deaths is incomplete. To fill this evidentiary gap, the Global Burden of Disease (GBD) study integrates different data sources with modelling assumptions to estimate incidence, prevalence, and disability-adjusted life years lost due to different NCDs including neoplasms, broad categories of cardiovascular disease (CVD), chronic respiratory diseases, DM and CKD [19, 20]. GBD methods have been extended for forward-projection as demonstrated by Malta et al., who projected NCD-related mortality trends in the Brazilian adult population through 2030 under four scenarios [21, 22]. However, to our knowledge no studies to date have attempted to project NCD mortality due to disruption of health services in crisis settings. Crises typically damage health and demographic information systems, even where, as in Gaza, they may be functional to begin with. This creates a serious gap when it comes to both tracking and anticipating the likely evolution of NCD morbidity and mortality, which may accordingly be overlooked in favour of other perceived priorities [23].

In the early phase of the war in Gaza, researchers from the London School of Hygiene and Tropical Medicine and the Johns Hopkins University’s Center for Humanitarian Health conducted a study to project excess mortality from different causes in the Gaza Strip up to August 2024 [24]. The projections covered five main modules: traumatic injury, infectious diseases, maternal, and neonatal, stillbirths, and NCDs, with malnutrition analysed as an underlying cause of mortality.

This paper describes the methods and findings of the model used to project NCD mortality under defined scenarios and outlines areas for further improvement in future applications. The study serves as a starting point for developing more comprehensive and accurate models to estimate future excess NCD mortality in humanitarian crisis settings.

## Methods

### Diseases included in the analysis

The analysis focused on specific NCDs with significant impact in Gaza: diabetes mellitus type 1 (DM1), cardiovascular diseases (CVD), chronic kidney diseases (CKD), and the most prevalent and highest-burden forms of cancer in Gaza (colorectal cancer, lung cancer, and breast cancer). Other NCDs were excluded due to limited pre-October 2023 baseline mortality data or insufficient information on survival rates with and without treatment. Table 1 lists NCDs included and excluded from the analysis, and corresponding reasons.

**Table 1:** List of non-communicable diseases included and excluded from Estimation

Disease (ICD-10† code)	Reason/additional notes
**Included**	
Diabetes mellitus type 1 (DM1) (E10)	A more pragmatic distinction could be insulin-dependent versus non-insulin-dependent DM. Obesity (E65 to E68) is considered an antecedent condition.
Cardiovascular diseases (CVD), including Ischaemic heart disease (including myocardial infarction, I21 to I25) Cerebrovascular diseases including stroke (I60 to I69) (this includes both ischaemic and haemorrhagic stroke, modelled separately due to their different survival: see below)	CVD is treated as an outcome of hypertension (and DM). As such, primary hypertensive disease (I10) is excluded from analysis.
Chronic kidney disease (CKD) (N18)	Only the most advanced stages of CKD, i.e. those requiring haemodialysis, is considered in this analysis. People requiring peritoneal dialysis are excluded.
Neoplasms (cancer), including Colorectal cancer (C18 to C21) Lung cancer (C34) Breast cancer (C50)	Highest-burden forms of cancer in Gaza (50).
**Excluded**	
Diabetes mellitus type 2 (DM2) (E11)	Insufficient published evidence found on survival with and without treatment.
Other forms of cardiovascular disease (CVD), including Rheumatic heart disease (I01-I09) Hypertensive diseases (I11 to I13) Inflammatory heart disease (I30-33, I38, I40, I42) Heart failure (I50) Diseases of arteries, arterioles and capillaries (I70 to I74)	Baseline mortality data for Gaza did not allow for unequivocal identification of these CVD causes. Furthermore, insufficient evidence was found on survival with and without treatment.
Chronic lower respiratory diseases, including Emphysema (COPD) (J43) Other chronic obstructive pulmonary disease (J44) Asthma (J45-J46)	Insufficient evidence found on survival with and without treatment.
Neoplasms (cancer), including Leukaemia (C91 to C95) Thyroid cancer (C73) Rarer cancers	Though leukaemia and thyroid cancer are also among the highest-burden cancers in Gaza [25, 26], we could not identify sufficient evidence on their survival with and without treatment.

† International Classification of Diseases, version 10 [26]

### Study population and period

The analysis comprised the entire population of Gaza. We used the United Nations Population Fund (UNFPA) May 2023 estimates to obtain age-specific population denominators; these are based on the Palestine census held on 1 December 2017 and provide male and female estimates for the five-year strata 0-4yo (years old) to 75-79yo, as well as ≥ 80yo [27, 28]. We stratified estimates into age groups $\alpha$ presented in the Supplementary File (Table S1). We ignore natural population growth and reduction due to war-attributable deaths during the analysis period, since births (about 50,000 assuming the pre-war annual birth rate of ≈ 30 per 1000) and deaths (around 60,000 based on a ground survey, assuming a constant death rate [29]) would have nearly cancelled each other out. The analysis period consisted of what we refer to a four-month sub-period ‘to date’ (7 October 2023 to start of scenario projections, 6 February 2024) and a six-month projection horizon further divided into two three-month sub-periods (7 February to 6 May 2024; 7 May 2024 to 6 August 2024).

### General model

Briefly, we compute excess mortality for each NCD and projection scenario separately as the difference between (a) projected deaths under wartime conditions and (b) expected deaths, had no war occurred. Scenarios are distinguished by varying assumed ranges of treatment coverage. We estimate (a) by simulating age- and sex-specific cohorts of incident cases based on pre-war trends, dividing this cohort into a treated and untreated fraction based on assumed coverage, and applying a risk of dying if treated or untreated (based on estimates from the literature) that, for all NCDs other than DM1, also depends on the case’s longevity (time since onset); and (b) by forecasting the trend of pre-war mortality into the war period. For CVD specifically, we modify the model by introducing an immediate risk of dying during acute events (stroke, heart attack), if treated or untreated, with mortality risk thereafter modelled by tracking the surviving patient cohort, as above.

For each NCD $$u$$, we consider that excess deaths at any given time *t* (where $$dt = 1 $$ month) are the difference between deaths projected in the war period to date or the projection period (subscript ‘crisis’) and a counterfactual level of baseline mortality (subscript ‘base’) that would have been expected in the absence of the crisis:

1 $$\:{D}_{u,t,\:\text{excess}}={D}_{u,t\text{,crisis}}-{D}_{u,t\text{,base}}$$

We can express both the crisis and counterfactual deaths in terms of prevalent treated and untreated cases and their respective risks of death, as follows:

2 $$\begin{aligned} \:{D}_{u,t,\:\text{excess}} ={c}_{u,t\text{,crisis}}\underset{\tau\:=1}{\overset{\tau\:={\tau}_{\text{max}}}{\int\:}}{P}_{u,t,{\rm T}, \text{crisis}}{\mu\:}_{u, {\rm T},\sigma =1} \\ \quad +\left(1-{c}_{u,t\text{,crisis}}\right)\underset{\tau\:=1}{\overset{\tau\:={\tau}_{\text{max}}}{\int\:}}{P}_{u,t,{\rm T},\text{crisis}}{\mu\:}_{u,{\rm T},\sigma=0}\\ \quad - {c}_{u,t\text{,base}}\underset{\tau\:=1}{\overset{\tau\:={\tau}_{\text{max}}}{\int\:}}{P}_{u,t,{\rm T}, \text{base}}{\mu\:}_{u,{\rm T},\sigma=1}\\ \quad +(1-{c}_{u,t\text{,base}})\underset{\tau\:=1}{\overset{{\infty\:}\tau\:={\tau}_{\text{max}}}{\int\:}}{P}_{u,t,{\rm T},\text{base}}{\mu\:}_{u,{\rm T},\sigma=0} \end{aligned}$$

Here, denotes treatment coverage, i.e. the proportion of cases who receive appropriate acute and follow-up care. Because the risk of dying for the NCDs analysed is not time-independent (see below), we track prevalent cases at time according to their time since onset $\tau$. Prevalent cases accordingly experience a $\tau$-specific hazard of death $(\mu)$, whose values vary according to whether a case is treated $(\sigma = 1)$ or untreated $(\sigma = 0)$. Thus, the sum of deaths per time step is the integral of mortality over the range of $\tau$.

For completeness, we recognise that, in addition to treatment disruptions, both the incidence (and thus prevalence) and case-fatality of some NCDs may be modulated by crisis-emergent risk factors, including disruptions in the management of antecedent conditions, as summarised in Table 2. As, such $\:{P}_{u,t\text{,crisis}}\sim{\phi\:}_{\lambda\:,\text{u},t}{P}_{u,t\text{,base}}$ and $\:{\mu\:}_{u,t\text{,crisis}}\sim{\phi\:}_{\mu\:,\text{u},t}{\mu\:}_{u,t\text{,base}}$, respectively, where $\:\phi\:$ denotes a relative risk (RR) of incidence ($\:\lambda\:$) or case-fatality ($\:\mu\:$) capturing all crisis effects. For this initial application of the model over what was a fairly short timeframe relative to the average evolution of many NCDs, we made a simplifying assumption that any changes in incidence attributable to the crisis would have had a modest effect on excess mortality due to the long-term progression of the NCDs being analysed, and thus set $\:{\phi\:}_{\lambda\:,\text{u},t}=1$. Similarly, we made a simplifying assumption that all excess case-fatality risk would solely be due to reduced coverage of acute and long-term treatment, i.e. $\:{\phi\:}_{\mu\:,u,t}=1$.

**Table 2:** Factors that May affect the risk of NCD incidence and case-fatality (other than disrupted treatment)

Effect	Risk factor				
	Stress and worsening mental health	Changes in dietary intake and diversity	Disrupted management of antecedent conditions	Exposure to smoke and dust from explosions, debris, inappropriate cooking fuel	Exposure to cold temperatures
Increased incidence	CVD, DM2, cancer	CVD (through hypertension)	CVD (through hypertension, DM2)		
Increased disease severity and thus case-fatality	All	CVD, DM1, DM2, CKD		COPD, asthma	COPD, asthma

We also assumed that the age distribution of deaths would not be affected by the crisis, i.e. $\:{p}_{a,\:u,\text{base}}={p}_{a,\:u,\text{crisis}}$, and distributed deaths accordingly. This assumption is not strictly correct: as treatment coverage decreases and survival accordingly shortens, the age distribution of excess mortality would shift towards younger populations. However, we verified through simulation that any resulting bias would have been negligible given the short analysis period (data not shown).

### Model parameterisation

#### Pre-war mortality by disease

To quantify pre-war age-specific CKD, ischaemic heart disease, and cerebrovascular disease mortality, we extracted Ministry of Health (MoH) 2022 data from the West Bank, since the latter applied a consistent ICD-10 classification [30], and scaled these to Gaza based on the ratio of West Bank to Gaza population size. We assumed that strokes were 50% ischaemic and 50% haemorrhagic [31, 32]. For cancer, we combined data from the International Agency for Research on Cancer and the Palestinian MoH [30, 33]. Generally, the resulting figures are similar in relative mortality terms to estimates for Lebanon and do not exceed European levels [34]. We also computed the mean age-specific proportion of deaths from disease $\:u$ ($\:{p}_{a,\:u,\text{base}})$ from the same sources.

We fitted a generalised linear model with Poisson distributional assumption and year as the single predictor to the annual count of deaths due to each NCD in the pre-war period, based on years of data availability (2021–2022 for ischaemic heart disease; 2017–2022 for all other NCDs). We used this model to forecast the counterfactual (no-crisis) number of deaths during 2023 and 2024, discretised to monthly increments, i.e. $\:{D}_{u,t\text{,base}}$.

We assumed that baseline DM1 mortality was negligible, plausibly due to the universal coverage of insulin treatment among recognised cases. Robust data on the number of prevalent cases of DM1 at the start of the war were however available from UNRWA and the MoH in Gaza [35, 36]. Therefore, we applied case-fatality hazards (see below) to this prevalent pool over the war period to date and the projection period based on assumed treatment coverage. We ignored incident DM1 cases since the war’s start as these would have been numerically small (≈ 3% out of a mean 17 DM1 plus DM2 incident cases per month pre-war, or about 6 DM1 cases annually).

#### Mortality hazard functions

For CVD, we considered that there is an immediate risk of death $\:{\mu\:}_{\text{acute}}\:$following the first acute presentation of the condition (e.g. stroke or heart attack). Conditional on surviving the acute event $\:\left(1-{\mu\:}_{\text{acute}}\right),\:$case-fatality during any subsequent time step since initial case onset is modelled as a time-dependent mortality hazard function $\:{\mu\:}_{u,\tau\:}$ (see below). While we recognise that patients often experience further acute events (e.g. repeat strokes), given the varying timing of such events we considered that population-level cohort studies of survival adequately capture the average risk of dying since onset. For other NCDs, we omitted the acute presentation of disease, again considering that population-level cohort studies capture how mortality changes as a function of $\:\tau\:$.

To parameterise $\:{\mu\:}_{u,\tau\:}$ with and without treatment, we reviewed published cohort studies describing both acute (for CVD only) and long-term survival (with at least three years of follow-up), identified through a non-systematic literature search and through contact with subject experts. We extracted from each study the proportion of patients surviving up to study-reported time points, out of the total at the start of follow-up. For cancer specifically, the West Bank’s MoH cancer registry provided informative data on survival with and without treatment based on follow up with cancer patients in the West Bank during 2017 to 2021 for four main cancer types (breast cancer, colorectal cancer, lung cancer and lymphoma) [37]. Analysis cohorts included patients who received surgical care only, and those who received chemotherapy and/or radiotherapy instead or in addition. We computed survival functions comparing patients who received surgical care only versus no care. We fitted candidate survival distributions to observations extracted from the literature, including negative-exponential, Weibull, log-normal and log-logistic, selecting the best-fitting based on maximum likelihood and conditioning on acute event survival for CVD. Where alternative studies were available, we fitted functions to either, to provide an upper and lower bound.

The following decay distributions were retained, depending on the NCD:

- **Log-normal**: $\:{\mu\:}_{\tau}=\frac{{f}_{\mathcal{N}}\left(\frac{\text{ln}\tau\:-\mu\:}{\sigma\:}\right)}{1-{F}_{\mathcal{N}}\left(\frac{\text{ln}\tau\:-\mu\:}{\sigma\:}\right)}$, where$\:{\:f}_{\mathcal{N}}$ is the probability density function of the standard normal distribution and $\:{F}_{\mathcal{N}}$ is the cumulative distribution function of the standard normal distribution;
- **Log-logistic**: $$\:{\mu\:}_{\tau\:}=\frac{\beta\:{\left(\frac{\tau\:}{\alpha\:}\right)}^{\beta\:-1}}{\alpha\:(1+{\left(\frac{\tau\:}{\alpha\:}\right)}^{\beta\:})}$$;
- **Exponential**: $$\:{\mu\:}_{\tau\:}=\lambda$$.

**Fig. 1:**
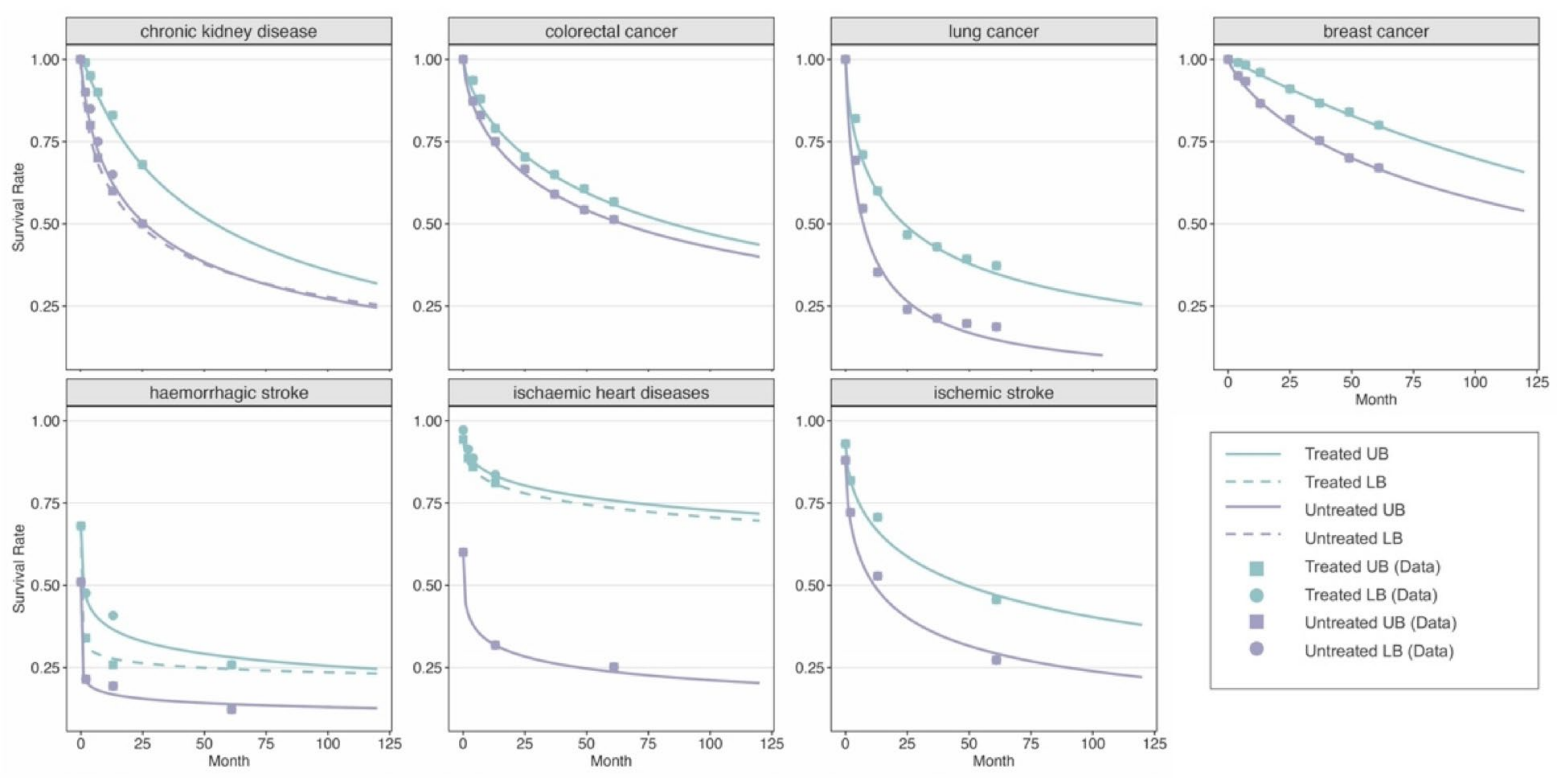
Survival fits for chronic kidney disease [38–43] colorectal [44], lung [44], breast cancer [44], haemorrhagic stroke [45, 46] ischaemic heart diseases [47–50] ischaemic stroke [51]. LB = lower bound; UB = upper bound

Parameters of each fitted survival function are provided in the Supplementary File, Table S2. We assumed age independence. Survival function fits are shown in Fig. 1.

#### Projection scenarios

We developed three scenarios informed by phone consultations with experts and consisting of alternative levels of population displacement, intensity and typology of military attacks, occurrence of any cessation of hostilities, functionality of healthcare services, and the ability of humanitarian actors to operate. In summary, the scenarios are as follows:

- **Ceasefire**: A permanent ceasefire is agreed, although a blockade with border checks remain. The majority of people remains in shelters because of large-scale destruction of residential buildings. Logistics improve and humanitarian assistance increases meeting minimal requirements, including for water, hygiene, and sanitation. Food provision conditions improve as well. Basic healthcare services are provided with an increase in medical supplies.
- **Status quo**: Humanitarian pauses of about 5–7 days are agreed. Aerial bombing and ground offensives continue across Gaza. Population displacement persists. There is a moderate increase in humanitarian assistance, but this is limited by military restrictions. Water and fuel are insufficient. Health service functionality is at low levels.
- **Escalation**: The military campaign focusses on crowded areas in southern Gaza but also persists elsewhere. There is large-scale further displacement to open areas and crowded shelters. Insecurity for humanitarian and health workers results in fewer services in fewer locations. Water, food, and fuel are very scarce. The majority of hospitals are partially or fully non-functional with limited access to care for the population.

More details on the scenarios are given in the Supplementary File (Table S3). Quantitatively, the scenarios are distinguished by different treatment coverage levels. We assumed that pre-war essential treatment coverage was 90–100%. While this does not equate to optimal treatment [52], it reflects the widespread availability of public health services including primary and secondary care in Gaza mainly through the MoH and UNRWA [53]. Based on pre-war data, expert knowledge, and conversations with health actors within Gaza, we considered it likely that the majority of NCD cases would have had access to essential treatment [52].

Data on pre-war NCD burden were primarily sourced from the Gaza MoH’s annual reports, available from 2017 to 2022. Where such data were not available, we used information from the occupied Palestinian territories as a whole from 2010 to 2022 or from the West Bank for cancer-related indicators from 2017 to 2021. For data related to treatment coverage during the ongoing war, we searched through Reliefweb (https://reliefweb.int/) for relevant grey literature and regular MoH emergency reports [54]. The reports highlighted, in particular, the acute shortage of insulin [55], the non-functionality of cancer hospitals [15] and a number of patients with CKD treated during the war [21, 22].

We reviewed this information to quantify ranges for the likely availability of treatment services required for management of different NCDs, both in the war period to date and over the projection period, the latter as scenario assumptions. These ranges are presented in the Supplementary File, Table S4. Specifically, we assumed that specialised services such as cancer care and haemodialysis would recover slowly during the ceasefire phase due to the destruction of key specialised care facilities and equipment and departure or death of clinical and allied health professionals needed to administer specialised NCD care. We assumed that humanitarian actors would not be able to offer cancer care even in a ceasefire scenario, and that patients would mostly not be allowed outside Gaza.

#### Model implementation

For each NCD, we simulated monthly incident cohorts of cases from January 2000 to the end of the projection period. We assumed a constant mean monthly incidence $\:{I}_{u,a,t,base}$, calibrated to fit observed mortality data as follows: for each of 1000 simulation runs, we sampled a random number of incident cases from a Poisson distribution centred at the above mean and computed the mean of simulated baseline deaths $\:{D}_{u,a,t\text{,base}}^{{\prime\:}}$ over the course of the convergence phase. A multiplier constant, $\:{k}_{u,a}$, was then used to scale $\:{I}_{u,a,t,base}$ so that $\:{D}_{u,a,t\text{,base}}^{{\prime\:}}={D}_{u,a,t\text{,base}}\:$.

We propagated uncertainty in treatment coverage and survival by sampling from uniform distributions of these parameters during each run (for survival, only where lower and upper bounds were available: see Table S2). Lastly, during each run we tracked both the ‘crisis-exposed’ cohorts (i.e. featuring the war period to date and scenario-projected treatment coverage) and cohorts experiencing counterfactual conditions (i.e. the pre-war treatment coverage): the difference in total deaths between these cohorts is these cohorts is $\:{D}_{u,a,t,\:\text{excess}}$. After simulating 1000 runs, we computed the mean and 95th percentile interval of outcomes. All analyses were done in R software [56] or Python.

#### Ethics approval and data availability

The study received approval from the London School of Hygiene & Tropical Medicine Research Ethics Committee and the Johns Hopkins Bloomberg School of Public Health Institutional Review Board. An open repository of data and analysis code is available on the GitHub platform (https://github.com/Gaza-projections/gaza_projections/tree/main/gaza_NCDs).

## Results

We estimated 1,479 excess deaths due to NCDs from the onset of war in October 2023 to early February 2024 (just before the projection period of the study), most attributable to ischaemic heart disease (Table 3). Over the period from February to August 2024, we projected 1,680 excess deaths in a ceasefire, 2,480 excess deaths in the status quo, and 2,676 excess deaths in an escalation scenario. Table 4 shows the breakdown by NCD and scenario.

**Table 3:** Projected excess mortality due to non-communicable diseases, by category, before the study projection period

Disease	Excess deaths (7 October 2023 to 6 February 2024)
Ischaemic heart disease	1,242 (1,114 to 1,376)
Chronic kidney disease†	33 (13 to 58)
Diabetes mellitus type 1‡	27 (0 to 71)
Stroke (haemorrhagic and ischaemic)	115 (45 to 196)
Cancer (lung, colorectal, breast)	62 (20 to 117)
Total	1,479 (1,192 to 1,818)

Values are the mean estimate and the 95% uncertainty interval

† Includes only people in need of haemodialysis. ‡ Other than through cardiovascular disease events

**Table 4:** Projected excess mortality due to non-communicable diseases, by category, and scenario during the six-month projection period (7 February to 6 August 2024)

Disease	Projection sub-period	Scenario		
		Ceasefire	Status quo	Escalation
Ischaemic heart disease	Months 1 to 3	776 (685 to 867)	992 (893 to 1,091)	1,044 (942 to 1,145)
	Months 4 to 6	651 (569 to 739)	962 (863 to 1,062)	1,006 (902 to 1,111)
	Total	1,427 (1,254 to 1,606)	1,954 (1,757 to 2,153)	2,050 (1,845 to 2,256)
Chronic kidney disease†	Months 1 to 3	21 (5 to 38)	34 (16 to 57)	39 (20 to 63)
	Months 4 to 6	15 (2 to 33)	32 (14 to 55)	38 (18 to 61)
	Total	36 (7 to 70)	67 (29 to 112)	77 (37 to 124)
Diabetes mellitus type 1‡	Months 1 to 3	18 (0 to 53)	97 (53 to 161)	141 (79 to 212)
	Months 4 to 6	18 (0 to 51)	95 (48 to 153)	130 (76 to 198)
	Total	36 (0 to 104)	191 (101 to 313)	271 (154 to 410)
Stroke (haemorrhagic and ischaemic)	Months 1 to 3	70 (22 to 131)	92 (37 to 155)	99 (40 to 164)
	Months 4 to 6	59 (12 to 117)	88 (33 to 153)	91 (37 to 162)
	Total	129 (34 to 247)	180 (71 to 308)	191 (77 to 326)
Cancer (lung, colorectal, breast)	Months 1 to 3	30 (6 to 71)	46 (12 to 88)	45 (12 to 90)
	Months 4 to 6	23 (−4 to 57)	43 (8 to 86)	42 (7 to 84)
	Total	53 (2 to 128)	89 (20 to 174)	87 (19 to 173)
Total	Months 1 to 3	915 (718 to 1,159)	1,261 (1,011 to 1,552)	1,368 (1,093 to 1,674)
	Months 4 to 6	766 (579 to 996)	1,219 (966 to 1,509)	1,308 (1,039 to 1,616)
	Total	1,680 (1,297 to 2,155)	2,480 (1,977 to 3,061)	2,676 (2,132 to 3,290)

Values are the mean estimate and the 95% uncertainty interval

†Includes only people in need of haemodialysis. ‡ Other than through cardiovascular disease events

**Fig. 2:**
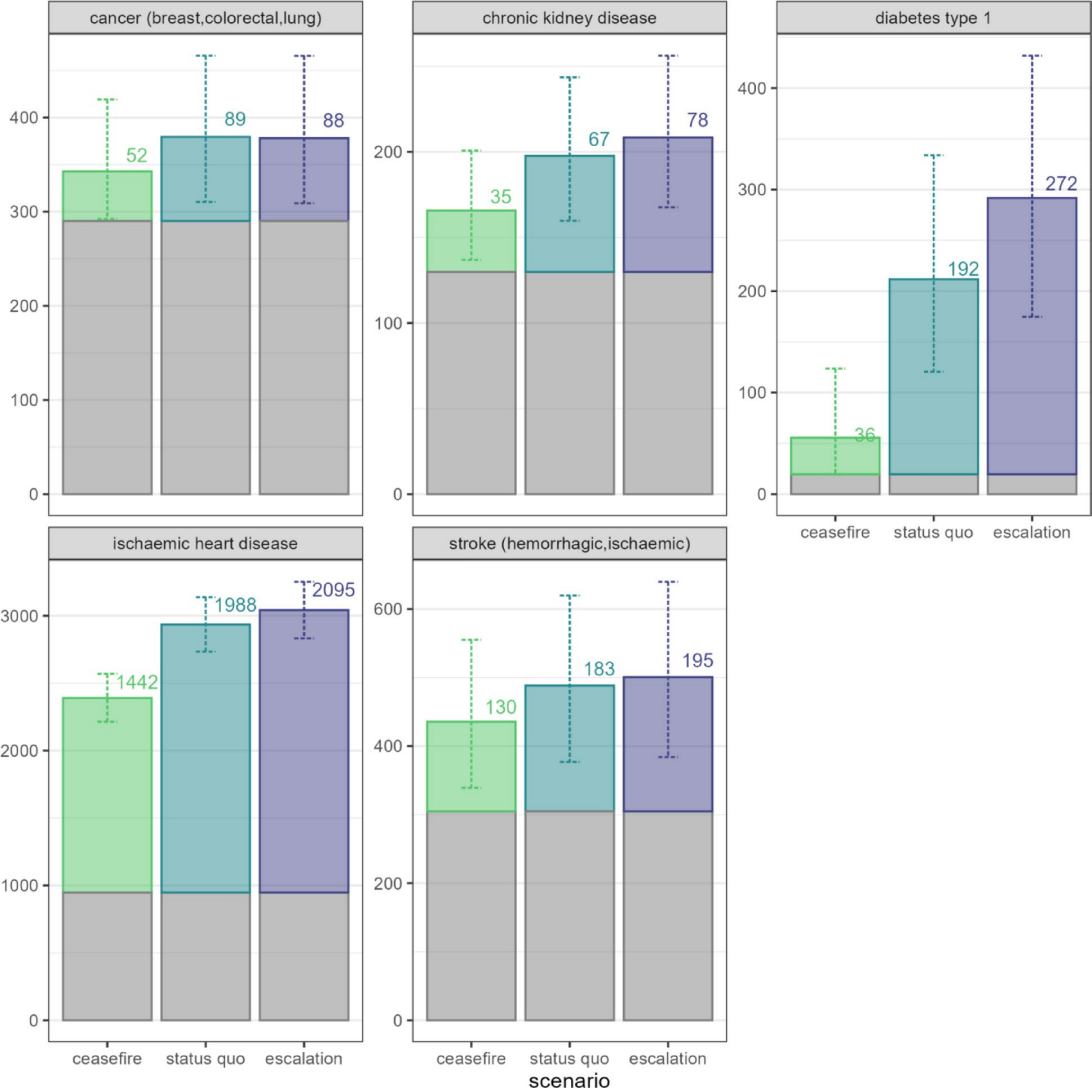
Projected number of deaths due to specific high-burden NCDs, by scenario over the six-month projection period. Counterfactual no-war deaths are shown in grey, and scenario-specific excess deaths in colour). Numbers indicate the point estimate, and vertical bars the 95% uncertainty interval

Figure 2 shows the projected numbers of deaths among patients with NCDs for each scenario over six months (7 February to 6 August 2024), showing the expected counterfactual deaths based on the pre-war baseline and excess deaths due to reduced treatment coverage. Ischaemic heart disease, DM1 and stroke were the largest contributors to projected mortality, in that order.

Across the three scenarios, most deaths were projected to occur among individuals aged *≥* 50yo (91%, 88% and 86% in the ceasefire, status quo and escalation scenarios, respectively). Few deaths were expected to occur among those aged 20 to 49yo, and very few for those 0 to 19yo (Table 5).

**Table 5:** Projected excess death toll (mean and 95% uncertainty interval) due to non-communicable diseases, by age, scenario, and period

Age	Period	Scenario		
		Ceasefire	Status quo	Escalation
0mo to 9yo	Months 1 to 3	1 (0 to 3)	7 (3 to 10)	9 (5 to 13)
	Months 4 to 6	1 (0 to 3)	7 (2 to 10)	9 (5 to 13)
	Total	2 (0 to 7)	12 (7 to 21)	18 (10 to 28)
10 to 19yo	Months 1 to 3	4 (1 to 8)	14 (8 to 23)	19 (11 to 29)
	Months 4 to 6	3 (0 to 8)	14 (7 to 22)	18 (11 to 28)
	Total	6 (1 to 16)	26 (15 to 44)	38 (22 to 75)
20 to 29yo	Months 1 to 3	8 (4 to 15)	22 (14 to 35)	30 (18 to 44)
	Months 4 to 6	7 (3 to 14)	22 (13 to 33)	28 (18 to 41)
	Total	15 (6 to 29)	44 (26 to 68)	58 (36 to 85)
30 to 39yo	Months 1 to 3	16 (11 to 25)	31 (21 to 44)	38 (25 to 52)
	Months 4 to 6	14 (8 to 22)	30 (20 to 42)	35 (24 to 49)
	Total	30 (19 to 46)	61 (41 to 86)	73 (50 to 100)
40 to 49yo	Months 1 to 3	51 (37 to 71)	83 (62 to 111)	96 (71 to 126)
	Months 4 to 6	43 (30 to 61)	81 (59 to 107)	91 (67 to 120)
	Total	94 (67 to 132)	164 (120 to 217)	188 (138 to 246)
50 to 59yo	Months 1 to 3	118 (93 to 151)	166 (132 to 205)	180 (143 to 222)
	Months 4 to 6	99 (74 to 129)	159 (125 to 199)	172 (135 to 213)
	Total	217 (167 to 281)	325 (257 to 404)	352 (278 to 435)
60 to 69yo	Months 1 to 3	176 (140 to 220)	236 (192 to 287)	253 (206 to 306)
	Months 4 to 6	146 (112 to 188)	227 (183 to 278)	241 (194 to 294)
	Total	322 (252 to 408)	464 (375 to 565)	494 (400 to 600)
70 to 79yo	Months 1 to 3	223 (177 to 278)	293 (239 to 353)	311 (254 to 374)
	Months 4 to 6	186 (142 to 238)	283 (228 to 344)	297 (241 to 361)
	Total	409 (320 to 515)	576 (467 to 697)	609 (495 to 735)
≥ 80yo	Months 1 to 3	318 (256 to 387)	409 (340 to 485)	431 (359 to 509)
	Months 4 to 6	267 (209 to 334)	398 (328 to 475)	416 (344 to 497)
	Total	585 (466 to 722)	807 (669 to 960)	847 (703 to 1,005)

**Fig. 3:**
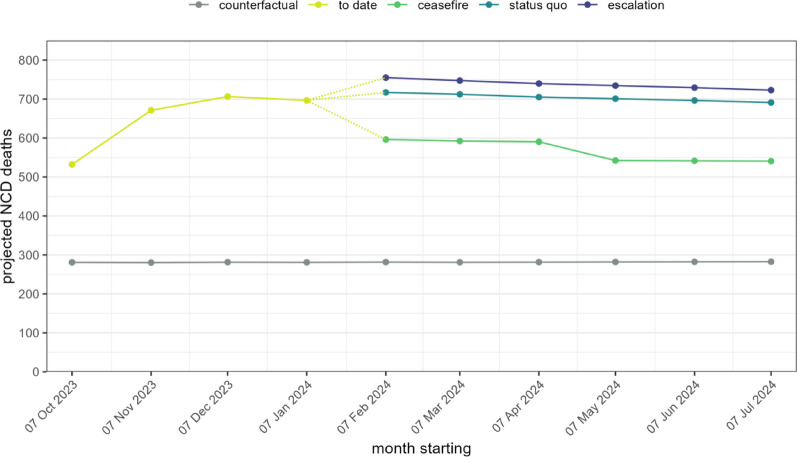
Monthly projected deaths due to all NCDs included in the analysis. For clarity only the point estimates are shown

In relation to expected counterfactual mortality, DM1 and ischaemic heart disease showed the greater relative increases during the projection period (Fig. 2). A mean of 281 deaths per month due to all NCDs combined was estimated under counterfactual assumptions, while projected deaths rose steeply during the period to date and peaked at 755/month under the escalation scenario (Fig. 3), corresponding to monthly mortality ratios (projections to counterfactual) between 1.9 and 2.5 during the period to date, and ranging between 1.9 and 2.7 depending on the scenario.

## Discussion

We present an initial application of a modelling approach to project NCD mortality in crisis settings. In Gaza, where the health system was systematically damaged by Israeli military forces, we project substantial excess mortality, particularly from CVD and translating to a doubling of more of deaths relatively to the counterfactual baseline. This modelling approach is potentially generalisable to other humanitarian responses as an evidence-based tool to project NCD excess mortality and thereby support decision-making. For example, the substantial mortality expected from DM1 suggests a need to systematically include measures such as insulin cold chain resilience and rapid identification of insulin-dependent patients within crisis preparedness and the initial package of humanitarian health interventions. However, robust baseline data and an understanding of the crisis, its affected population’s epidemiological profile and the functionality and performance of the health system, are essential to develop plausible scenarios and estimates to inform context-adapted humanitarian responses.

NCDs were the leading cause of death in 2022 in the Gaza Strip [57], and the model suggests that the first 10 months of war exacerbated NCD mortality considerably. The primary cause of excess mortality within the NCD model was ischaemic heart disease, specifically myocardial infarctions, which would primarily be explainable through lack of access to cardiac catheterization, interventional cardiologists, and thrombolytics. Excess mortality for stroke would have arisen from the lack of availability of operating rooms and neurosurgeons for haemorrhagic stroke, and insufficient access to thrombolytics and neuro-interventionists for ischaemic stroke. Excess mortality from DM1 would have resulted from disruption in insulin supply, while CKD deaths could have arisen from various causes including missed haemodialysis sessions in patients with end-stage renal disease.

In the ceasefire scenario, projected excess NCD mortality was lower than the other scenarios, reflecting the improvement over time in health service access and quality, including resumption of referrals for treatment outside of Gaza. Unsurprisingly, across the three scenarios, most deaths were projected to occur among individuals aged *≥* 50 years. We are aware of two ground mortality surveys conducted to date in Gaza, and which measured deaths not due to war-related injury, providing a possible comparison with our projections. Spagat et al. [29] estimate 8540 (95%CI 4,540 to 12,500) such deaths between 7 October 2023 and 5 January 2025, but without disaggregation by cause: assuming a constant rate, this estimate would scale to about 5700 (3000 to 8300) over our period of analysis (7 October 2023 to 6 August 2024). By contrast, under the status quo scenario, we projected 3959 NCD excess deaths, 5782 due infections [58] and 315 due to maternal and neonatal causes [24], totalling 10,056. If the ground survey is accurate, our NCD projections may be somewhat higher than reality. A second survey, covering only Médecins Sans Frontières staff and their families, finds a proportion of deaths due to causes other than war-related injury similar to ours and Spagat et al.’s, and reports that two thirds of NCD patients experienced treatment interruptions [59]. Further mortality studies may, in future, help to further validate our projections, though we stress that we set out to *project* mortality based on scenarios, not *predict* its future value.

### Model limitations

The NCD conditions included in our analysis accounted for about 70–80% of all NCD deaths pre-war according to MoH data. However, we omitted from the analysis key high-burden NCDs, including diabetes mellitus type 2 (DM2), chronic obstructive pulmonary disease, and other types of common cancers in Gaza; these conditions may account for a larger burden of disease in other settings where the model might find application, resulting in considerable underestimation of NCD excess deaths. While DM2 mostly leads to death via CVD and thus may largely be captured by CVD MoH mortality figures, some DM2 deaths occur due to other complications including diabetic ketoacidosis and hyper-osmolar hyperglycaemic state, neither of which are captured by our analysis. Disease omission was mostly due to insufficient information on NCD survival, especially without treatment. Given the limited study timeframe, we could only conduct non-systematic keyword searches, supplemented by engagement with subject experts to identify additional suitable evidence. Studies on which model parameters are based may not be representative of the profile of pre-war patients in Gaza due to differences in demographics, clinical presentation, and specific treatment regimens, resulting in under- or over-estimation of mortality. For cancer specifically, we calculated survival based on surgically treated cases, omitting the better outcomes experienced by the fraction of cases who would have had continued access to radio- and chemotherapy in the absence of a war: this may have resulted in some under-estimation of excess cancer mortality.

We assumed that treatment coverage was binary, while patients may in fact be on a spectrum between excellent treatment access, minimal delays and high adherence, on the one hand, and no treatment at all, on the other, with interruptions being a commonly described outcome in crisis settings [60]. This dichotomisation is difficult to avoid in a context of scarce evidence on survival but could have resulted in some bias if cases with partial treatment were relatively more similar to either extreme.

In this model we assumed that treatment coverage and survival are age-independent, but this may not hold true. If these parameters are negatively correlated with age-specific incidence (i.e. the age groups with highest incidence are also those with lowest coverage and survival), our analysis would underestimate mortality, and vice versa. Likewise, we did not model increases in NCD incidence and disease progression due to the war itself, resulting in further under-estimation of the excess. Such risks would plausibly play out over a longer timeframe (e.g. disruptions in treatment of hypertension would lead to progression to more severe stages and eventually a higher risk of CVD), but, as the crisis continues, their effect is likely to compound and would need to be explicitly accounted for by models. In addition, we were unable to stratify the projections by sex; however, this is essential since NCD-related mortality might differ between men and women.

Referrals outside Gaza were also common for conditions other than cancer before the war. For instance, the 5,864 MoH-reported referrals for cancer treatment outside Gaza in 2022 accounted for only 27% of 22,123 total referrals [57]; unsuccessful applications for permits to exit the Gaza Strip had a significant impact on mortality for cancer patients (hazard ratio 1.45) [37]. The sudden reduction in referrals for non-cancer NCDs during the war is not accounted for in our models.

The above limitations, taken together, bias our estimates towards considerable under-estimation of true excess mortality, and should thus be considered when interpreting our projections.

### Future directions

This study represents a first attempt to project excess mortality from NCDs in a live war setting. Projecting excess mortality through a quantitative model could provide critical evidence for advocacy on the plausible health impact of a humanitarian crisis on vulnerable populations; help key humanitarian decision-makers plan their responses and prioritise health threats appropriately [61]; and foster compliance with international humanitarian law [62], for example by guaranteeing safe access to patients and quality healthcare services. A modelling approach using the Lives Saved Tool has been utilised to project maternal and neonatal mortality resulting from insecurity in Sudan and the 2014 Ebola epidemic’s healthcare disruptions in Sierra Leone [63, 64].

However, generalisation of this model to other humanitarian responses will require improving on some of the above limitations, expanding the range of NCDs covered, and collaborative development and validation involving the NCD research community. We suggest that the main areas for improvement include:

- Conducting systematic literature reviews and, if these do not yield suitable evidence, structured expert elicitation to help quantify the model parameters of omitted diseases, especially values for NCD survival with and without treatment;
- Introducing more detail in the model, including on underlying causes, and different stages and treatment regimens of each NCDs, similar to the GBD approach [19, 20];
- Explicitly modelling the increase in NCD incidence and disease progression to severe stages as a function of disrupted treatment; for example, for CVD this may require modelling the progression of patient cohorts from healthy to successive stages of hypertension featuring escalating (age- and sex-specific) risks of acute CVD events and heart failure, as a function of whether patients are receiving hypertensive medication;
- Including additional, non-treatment factors that may affect the risk of NCD incidence and case fatality, such as the effect of malnutrition or worsened mental health (Table 2). Malta and colleagues used a comparative risk assessment to determine the risk factors’ contribution to mortality by NCDs through comparing the health outcomes with those that would have been seen in a counterfactual exposure group in which no individual was exposed [21, 22].
- Modelling co-morbidity between DM and hypertension and their effect modification for downstream conditions including CVD and CKD; for CKD this may entail progressing the population through different renal function stages including haemodialysis-reliant end-stage disease as function of the corresponding dynamics of DM-hypertension stage population classes.

## Conclusion

To our knowledge this study is the first to project the excess mortality due to NCDs in a war setting. The findings revealed increased mortality due to the disruption of healthcare services’ access, supply, and quality. The study also showed most projected deaths to occur among individuals aged *≥* 50yo, which emphasises the vulnerability of these groups in war settings. The relatively simplistic model used here likely underestimates actual excess mortality due to the omission of some diseases and increases in NCD incidence and disease progression due to factors other than disrupted treatment. This model could be improved to provide more accurate estimates and used in other contexts to inform decision-making.

## Supplementary Information

Supplementary Material 1

## Data Availability

An open repository of data and analysis code is available on the GitHub platform (https://github.com/Gaza-projections/gaza_projections/tree/main/gaza_NCDs).
