# Supplementary material for: Modelling excess mortality from non-communicable diseases during wartime: application to the Gaza Strip, occupied Palestinian territories
Source: Popul Health Metr. 2025 Nov 26;23:68. doi: 10.1186/s12963-025-00426-5 (PMC12659381; doi:10.1186/s12963-025-00426-5)
Supplement: Supplementary file 1 — Supplementary Material 1 [file 12963_2025_426_MOESM1_ESM.docx]

Modelling excess mortality from non-communicable diseases during wartime: Application to the Gaza Strip, occupied Palestinian territories

**SUPPLEMENTARY FILE**

Table S1. Pre-war Gaza population based on a UNFPA 2023 projection [1].

| **Age** | **Population** |
| --- | --- |
| <1 months | 5,810 |
| 1-11 months | 63,911 |
| 12-59 months | 267,336 |
| 5-9 years | 284,458 |
| 10-14 years | 277,192 |
| 15-19 years | 235,937 |
| 20-29 years | 383,776 |
| 30-39 years | 302,060 |
| 40-49 years | 181,617 |
| 50-59 years | 118,024 |
| 60-69 years | 67,854 |
| 70-79 years | 30,106 |
| 80-100 years | 8,463 |
| Total | 2,226,544 |

Table S2. Parameters of fitted survival functions, by disease and treatment status.

| **Disease** | **Treatment status** ($\sigma$) | **Error bound** | **Probability of acute phase survival** ($\mu_{\text{acute}}$) | **Distribution parameters for** $\boldsymbol{\mu}_{\boldsymbol{u}\mathbf{,}\boldsymbol{\tau}}$ (conditional on acute-phase survival for CVD) | | |
| --- | --- | --- | --- | --- | --- | --- |
| **Log-normal distributed** | | | | $\boldsymbol{\mu}$ | $\boldsymbol{\sigma}$ |  |
| Chronic kidney disease | 1 (yes) | single estimate | n/a | 4.00 | 1.68 |  |
|  | 0 (no) | lower | n/a | 3.14 | 1.58 |  |
|  |  | upper |  | 3.26 | 1.58 |  |
| CVD: myocardial infarction | 1 | lower | 0.94 | 8.10 | 5.19 |  |
|  |  | upper | 0.97 | 8.10 | 5.19 |  |
|  | 0 | single estimate | 0.60 | 2.90 | 4.50 |  |
| **Log-logistic distributed** | | | | $\boldsymbol{\alpha}$ | $\boldsymbol{\beta}$ |  |
| CVD: haemorrhagic stroke | 1 | lower | 0.68 | 0.70 | 0.12 |  |
|  |  | upper | 0.68 | 20.70 | 0.32 |  |
|  | 0 | single estimate | 0.60 | 0.24 | 0.18 |  |
| CVD: ischaemic stroke | 1 | single estimate | 0.93 | 63.17 | 0.58 |  |
|  | 0 | single estimate | 0.88 | 18.56 | 0.58 |  |
| Breast cancer | 1 | single estimate | n/a | 221.35 | 1.06 |  |
|  | 0 | single estimate | n/a | 146.60 | 1.06 |  |
| Colorectal cancer | 1 | single estimate | n/a | 84.41 | 0.73 |  |
|  | 0 | single estimate | n/a | 64.49 | 0.65 |  |
| Lung cancer | 1 | single estimate | n/a | 23.64 | 0.66 |  |
|  | 0 | single estimate | n/a | 7.15 | 0.66 |  |
| **Negative-exponential distributed** | | | | $\boldsymbol{\lambda}$ | | |
| Diabetes mellitus type 1 | 1 | single estimate | n/a | 0.001† | | |
|  | 0 | lower | n/a | 0.06 [2] | | |
|  |  | upper | n/a | 0.03 | | |

† Inverse of Gaza population life expectancy [3].

Table S3. Key characteristics of three scenarios.

| **Characteristic** | **Scenario** | | |
| --- | --- | --- | --- |
|  | **Ceasefire** | **Status quo** | **Escalation** |
| Occurrence and duration of any pauses/ceasefires | A permanent ceasefire occurs but Gaza continues to be under blockade, with ongoing border restrictions on transit of people and goods. | Two or three ‘humanitarian’ pauses each of about 5-7 days during the six-month projection period. | None. |
| Intensity and typology of military activity | None. | Aerial bombing and ground offensive continues including in South Gaza. Gaza is under de facto military control during the humanitarian pauses. | Aerial bombardment and ground operations increase in intensity, shifting focus to the crowded areas in the South of Gaza while also resuming in the North. |
| Population displacement | The population starts to return home, but the majority remain in shelters due to destroyed dwellings. | Displacement persists as people continue to move south. | Continued large-scale displacement, now into more open areas as shelters are full. |
| Humanitarian space and operational adaptation | - There is a large influx of humanitarian assistance that increases over time as logistics improve.  - The international community supports an emergency response.  - Challenges persist, but adaptive humanitarian efforts address evolving needs.  - WASH conditions improve in shelters and open areas, and food provision increases. | - Moderate but limited increase in aid, and humanitarian action constrained by military restrictions.  - Water and fuel continue to be at insufficient levels.  Some adaptation of humanitarian services in the South, e.g. by moving health services closer to internally displaced persons (IDPs). | - Insecurity for humanitarian and health workers worsens, resulting in fewer services and aid delivered in fewer locations.  - Water, food, and fuel available to the population remains very scarce. |
| Functionality and performance of health services | - Health services operate with improved and sufficient supplies, but specialised services remain inadequate due to loss of skilled health workers and equipment.  - Pathways of patient referral for treatment outside Gaza are gradually restored.  Humanitarian actors adapt their operations by shifting activities closer to the population, and large vaccination campaigns occur. | Functionality of health services remains at the current low levels, with limited supplies and quality of services remaining very constrained. | - More health facilities become partially or fully non-functional and many people are in areas where there were fewer health facilities to begin with.  - Most of the population has less access to care due to limited hospitals, clinics, and transportation to healthcare facilities. |

Table S4. Assumed ranges of treatment coverage for specific NCD-relevant treatment services, by scenario.

| **Period** | **Percentage of patients able to access haemodialysis** | | | **Percentage of the population with access to functional inpatient public departments able to administer emergency care for cardiovascular events** | | | **Percentage of patients with access to insulin** | | | **Percentage of cancer patients able to access treatment (surgery only)** | | |
| --- | --- | --- | --- | --- | --- | --- | --- | --- | --- | --- | --- | --- |
|  | CKD | | | CVD | | | DM1 | | | Cancer | | |
| Pre-war | 90-100% | | | 90-100% | | | 90-100% | | | 90-100% | | |
| To date |  | | | | | | | | | | | |
| 7 Oct 2023 to 6 Nov 2023 | 70-80% | | | 40-60% | | | 90-100% | | | 20-40% | | |
| 7 Nov 2023 to 6 Dec 2023 | 30-50% | | | 10-30% | | | 90-100% | | | 1-5% | | |
| 7 Dec 2023 to 6 Jan 2024 | 20-40% | | | 5-15% | | | 90-100% | | | 1-5% | | |
| 7 Jan 2024 to 6 Feb 2024 | 20-40% | | | 5-15% | | | 90-100% | | | 1-5% | | |
| Projection | Scenario† | | | | | | | | | | | |
|  | C | S | E | C | S | E | C | S | E | C | S | E |
| 7 Feb 2024 to 7 Apr 2024 | 40-60% | 15-20% | 5-10% | 20-40% | 5-15% | 1-5% | 90-100% | 70-80% | 60-70% | 1-5% | 1-5% | 1-5% |
| 7 May 2024 to 6 Aug 2024 | 50-70% | 15-20% | 5-10% | 30-50% | 5-15% | 1-5% | 90-100% | 70-80% | 60-70% | 1-5% | 1-5% | 1-5% |

†C = ceasefire; S = status quo; E = escalation. Values during the period to date (before projection) are the same as for the status quo projection scenario.

# References

1. UNFPA. State of Palestine - Subnational Population Statistics - Humanitarian Data Exchange. https://data.humdata.org/dataset/cod-ps-pse. Accessed 15 Feb 2024.

2. Brostoff JM, Keen H, Brostoff J. A diabetic life before and after the insulin era. Diabetologia. 2007;50:1351–3. https://doi.org/10.1007/s00125-007-0641-0.

3. World Bank Open Data: Life expectancy at birth. World Bank Open Data. https://data.worldbank.org. Accessed 14 Feb 2024.
